# LRGCPND: Predicting Associations between ncRNA and Drug Resistance via Linear Residual Graph Convolution

**DOI:** 10.3390/ijms221910508

**Published:** 2021-09-29

**Authors:** Yizhan Li, Runqi Wang, Shuo Zhang, Hanlin Xu, Lei Deng

**Affiliations:** School of Computer Science and Engineering, Central South University, Changsha 410083, China; downs5@csu.edu.cn (Y.L.); wrqsoftware@csu.edu.cn (R.W.); 8209180419@csu.edu.cn (S.Z.); xuhanlin@csu.edu.cn (H.X.)

**Keywords:** ncRNA, drug resistance, association prediction, graph convolution network, feature propagation

## Abstract

Accurate inference of the relationship between non-coding RNAs (ncRNAs) and drug resistance is essential for understanding the complicated mechanisms of drug actions and clinical treatment. Traditional biological experiments are time-consuming, laborious, and minor in scale. Although several databases provide relevant resources, computational method for predicting this type of association has not yet been developed. In this paper, we leverage the verified association data of ncRNA and drug resistance to construct a bipartite graph and then develop a linear residual graph convolution approach for predicting associations between non-coding RNA and drug resistance (LRGCPND) without introducing or defining additional data. LRGCPND first aggregates the potential features of neighboring nodes per graph convolutional layer. Next, we transform the information between layers through a linear function. Eventually, LRGCPND unites the embedding representations of each layer to complete the prediction. Results of comparison experiments demonstrate that LRGCPND has more reliable performance than seven other state-of-the-art approaches with an average AUC value of 0.8987. Case studies illustrate that LRGCPND is an effective tool for inferring the associations between ncRNA and drug resistance.

## 1. Introduction

Non-coding RNAs (ncRNAs) play special roles in the development, differentiation, and aging of cells. Numerous studies have shown that ncRNAs are widely involved in human pathological activities. They act as biomarkers to provide new targets for the treatment of diseases such as cancer [1]. Non-coding RNAs such as microRNAs (miRNAs), circular RNAs (circRNAs), and long ncRNAs (lncRNAs) have aroused great interest of researchers. miRNAs are short regulatory biomolecules that are involved in the post-transcriptional regulation of gene expression [2]. Compared with linear miRNAs, circRNAs [3] are more stable and may function as transporters or scaffolds [4]. They exert essential biological functions by acting as microRNA or protein inhibitors (“sponges”), regulating protein function, or being translated themselves [5]. lncRNA can play a role in regulating cooperating proteins [6]. piRNA (Piwi-Interacting RNA) has been relatively poorly studied compared to those three. piRNA can form a piRNA/PIWI complex with PIWI proteins to affect gene expression and mainly function to suppress the activity of transposons [7,8]. There are synergies among RNAs. For example, lncRNA can act as a molecular sponge of miRNA to regulate the expression of its target gene [9,10,11,12].

According to a statistical cancer report released by the American Cancer Society [13], it is estimated that there will be approximately 4950 new cancer cases and 1600 deaths due to cancer every day in the United States. Unfortunately, the development of drug resistance greatly increases the probability of recurrence and significantly reduces the cure rate. Drug resistance has become a major obstacle to clinical treatment.

With the development of sequencing technology, it has been reported that cancer resistance to treatment is related to mutations of the cell’s genome [14,15]. The instability of the genome may change the phenotype of the tumor and lead to drug resistance. Studies have shown that some ncRNAs, such as miRNAs, can act as rheostats to regulate protein output [16]. The abnormal expression of ncRNAs is not only associated with several diseases but also may promote drug resistance of cancer cells [17,18]. circRNA acts as a miRNA sponge and enhances the response of HCC (hepatocellular carcinoma) cells to chemotherapy with cisplatin [19]. lncRNA enhanced drug resistance in AML (acute myeloid leukemia) cells by inhibiting miR-186 [20]. Overexpression of miRNA-194 can make HCC cells more sensitive to sorafenib [21]. Increasing evidence suggests that drug resistance is affected by ncRNA. Exploring their interaction will provide new insights for improving the therapeutic effect.

The relationship between ncRNAs and drug resistance has been gradually discovered, and some databases already provide relevant data. The ncDR database [22] provides 135 compounds and 1050 ncRNAs. Additional information on compounds and ncRNAs, such as ncRNA genomic contexts, had also been added. NoncoRNA [23] covers the basic calculation of ncRNA, drugs, diseases, etc., and includes experimental detection techniques, drug response, and other information. However, existing knowledge is minimal compared to the unknown associations. Discovering possible relationships between ncRNAs and drugs is beneficial for understanding related drug resistance mechanisms and accelerating drug development. To some extent, the traditional biological experiments are difficult to be carried out due to the factors such as difficult control and high time cost. Computational methods are useful accelerators of this process, but very little work has been done in this area.

In recent years, association prediction methods have been greatly developed in Bioinformatics. GCMDR [24] established a three-layer latent factor model to predict miRNA-disease associations introducing features such as miRNA expression profile and drug PubChem substructure fingerprints into the model. Zhu et al. [25] utilized the matrix completion method. SDLDA [26] introduced singular value decomposition and ILNCRNADIS-FB [27] calculated the three-dimensional feature blocks to capture characteristics. In a different way, SAEMDA [28] extracts features through semantic similarity. In terms of the prediction of circRNA-disease associations, AE-RF algorithm [29] also integrates many information sources to obtain the depth features. DMFCDA [30] constructed a circRNA-disease matrix with explicit and implicit feedback to capture the non-linear features. Deng et al. [31] constructed a heterogeneous information network (HIN) containing multiple subnetworks. A great deal of research has focused on the microbe-disease association prediction. The KATZHMDA [32] introduced the Gaussian kernel to perform a complete and easy reconstruction of the microbe-disease relationship. The ABHMDA [33] is a strong classifier based on the existing model to achieve better self-adaptability. Liu et al. [34] used matrix decomposition based on neural networks to obtain nonlinear latent features to infer disease-related microbes. The NTSHMDA [35] successfully reduced the prediction error by assigning random walks according to different weights.

**Figure 1:**
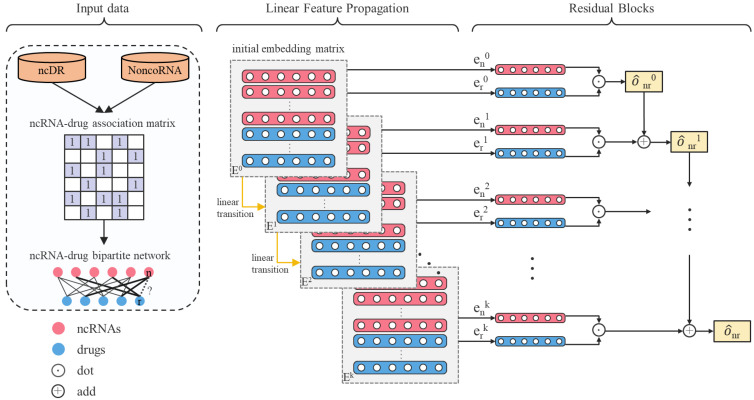
Flow chart of LRGCPND. $E^{0}$ denotes the embedding matrix in layer 0. $e_{n}^{0}$ and $e_{r}^{0}$ denote the embedding of ncRNA n and drug d in layer 0, respectively. LRGCPND contains three steps: aggregation, linear transition, and residual learning. In the feature aggregation step, we use the spectral rule to aggregate the features of neighboring nodes. After that, the linear transformation is adopted to speed up the forward propagation. Finally, we add a residual block to fuse the characteristics of low-layer nodes directly, attaining higher-layer potential features.

Although the above methods have achieved good results, some problems and shortcomings still hinder more comprehensive potential feature mining. The lack of relevant biological data and information leads to noise in the calculated features, which reduces prediction accuracy. Existing association predictions are more dependent on the existing similarities in the database. When the number of ncRNAs and diseases increases, the existing calculation models are difficult to draw conclusions efficiently, so they are not suitable for large-scale data sets. Therefore, these methods are not applicable when predicting the relationship between multiple ncRNAs and drug resistance. Although more and more ncRNA-drug resistance associations have been determined and existing databases provide relevant data, the existing knowledge is still very limited compared with the unknown potential associations. Here we propose an efficient approach based on a linear residual graph convolutional network, LRGCPND, which only employs ncRNA and drug resistance validated interactions. Initially, LRGCPND constructs a bipartite graph through the association network of ncRNA and drug resistance, where the edges represent the hidden interaction factors between the two types of nodes. The unconnected edges may have associations that are not obvious to identify. LRGCPND then fleetly aggregates the intrinsic characteristics of neighbor nodes in the former layer and performs the linear transition. After the specified number of iterations, it fuses the embeddings of previous convolutional layers through residual learning to favorably explore the interactions between ncRNA and drug resistance. LRGCPND achieves the best performance compared with the other advanced computational methods. Case studies of two anti-cancer drugs demonstrate the practical capability of LRGCPND. The flow chart of LRGCPND is shown in Figure 1.

## 2. Results and Discussion

### 2.1. Experimental Setup

To objectively and systematically evaluate the ability of LRGCPND and expedite comparison with other methods, we perform k-fold cross-validation (k-fold CV) on the collected dataset. All verified associations are randomly divided into k parts. Each part is picked as positive samples with an equal quantity of unlabeled samples as negative samples to form the testing set. Meanwhile, the equivalent operation is performed on the remaining k−1 parts to obtain the training set. This process ends after k iterations.

Even if there may be latent associations in the selected negative samples, since they account for a tiny proportion in the entire unverified sample set, the influence is negligible.

### 2.2. Evaluation Criteria

To observe intuitively and comprehensively, we measure the performance of models by widely adopted metrics, including AUC, AUPR, Accuracy (Acc.), Precision (P.), Recall (R.), and F1 scores, which are defined by the following formula:

(1) $$\mathrm{Accuracy}=\frac{\mathrm{TP}+\mathrm{TN}}{\mathrm{TP}+\mathrm{TN}+\mathrm{FP}+\mathrm{FN}}$$

(2) $$\mathrm{Precision}=\frac{\mathrm{TP}}{\mathrm{TP}+\mathrm{FN}}$$

(3) $$\mathrm{Recall}=\frac{\mathrm{TP}}{\mathrm{TP}+\mathrm{FN}}$$

(4) $$F1=\frac{2*\mathrm{Precision}*\mathrm{Recall}}{\mathrm{Precision}+\mathrm{Recall}}$$

TP and FP represent the number of correct and incorrect classifications in the related ncRNA-drug resistance pairs. In contrast, TN and FN represent the number of correct and incorrect classifications in the unrelated pairs. By adjusting the threshold, we can plot the receiver operating characteristic (ROC) curve and precision-recall (PR) curve and then calculate the area under the curves to get AUC and AUPR, respectively.

### 2.3. Performance Evaluation for LRGCPND

**Figure 2:**
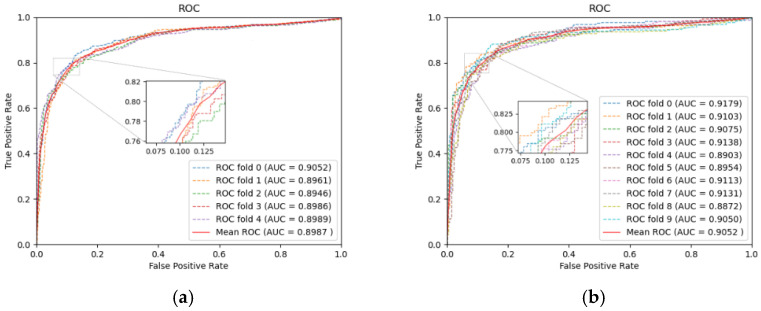
Performance of LRGCPND in five-fold CV and ten-fold CV, respectively. (**a**) ROC curves yielded by LRGCPND in five-fold CV. (**b**) ROC curves yielded by LRGCPND in ten-fold CV.

To evaluate the identification ability of our model, we performed five-fold and ten-fold CV on the dataset specified above. Table 1 lists the specific results in five-fold CV, and Figure 2 displays the ROC curves. In five-fold CV, the average values of AUC, AUPR, and Accuracy reach 0.8987, 0.9094, 0.8342, respectively. With the increasing size of the training set, training of the model will achieve a more thorough level. So, in ten-fold CV, the AUC increased to 0.9052. As seen from the above experimental results, LRGCPND can accurately and effectively identify potential ncRNAs related to drug resistance.

### 2.4. Effects of Parameters

**Figure 3:**
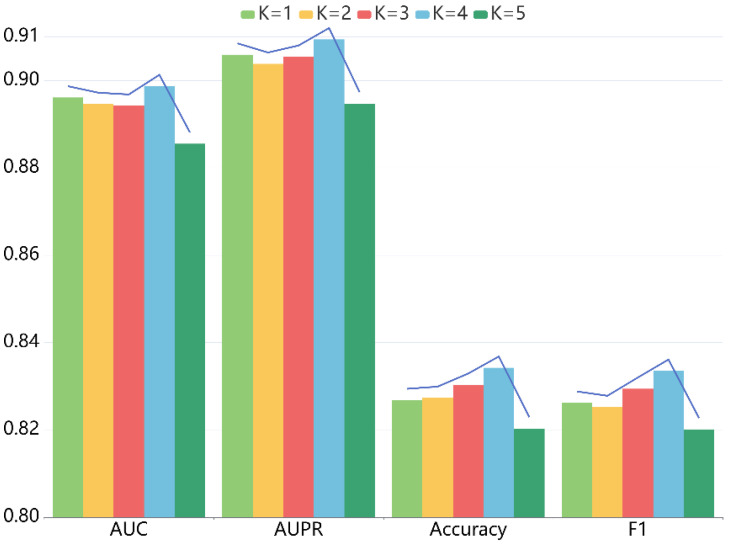
Effects of depth *K* on the performance of LRGCPND.

For LRGCPND, there are two crucial parameters: the depth of propagation and the dimension of embedding, which influence the prediction capability. For one thing, we explored the impact of layer depth K, following the settings of other parameters constant. When K ranges from 1 to 5, we performed five-fold CV. Table 2 lists the detailed data, and Figure 3 is the trend chart of different indicators. Our model achieves the best performance when K is equal to 4.

**Figure 4:**
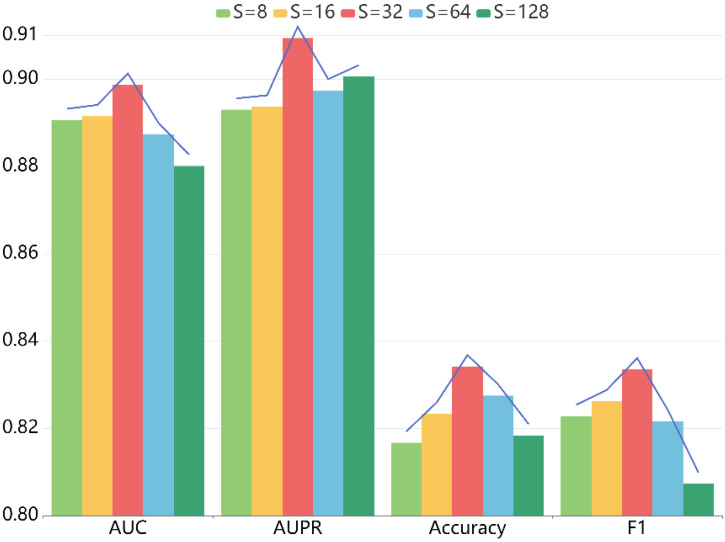
Effects of embedding size *S* on the performance of LRGCPND.

For another thing, the embedding dimension S also has a critical role. When setting the value of S to 8, 16, 32, 64, 128 sequentially, we conducted five-fold CV to measure the impact on the prediction ability of our model. Table 3 shows the detailed statistics, and Figure 4 indicates the trend of diverse metrics. From the results, we can conclude that when S varies from 8 to 128, the performance first monotonically improves. That is because the larger embedding dimension enhances the expressivity of LRGCPND to a certain extent. When S is 32, it reaches the optimum. Then as S increases, it starts to produce adverse effects on the performance.

In other experiments, we employ the optimal values obtained above as the default of model parameters.

### 2.5. Comparison with Other Approaches

Since inferring ncRNA-drug resistance interactions is a relatively new area, no researchers have proposed relevant solutions already. Nonetheless, reviewing other association prediction methods in bioinformatics still provides significant references for the performance of our model. To further assess the effectiveness of LRGCPND, we compared it with seven advanced approaches in directions of lncRNA-disease, circRNA-disease, and microbe-disease.

**Figure 5:**
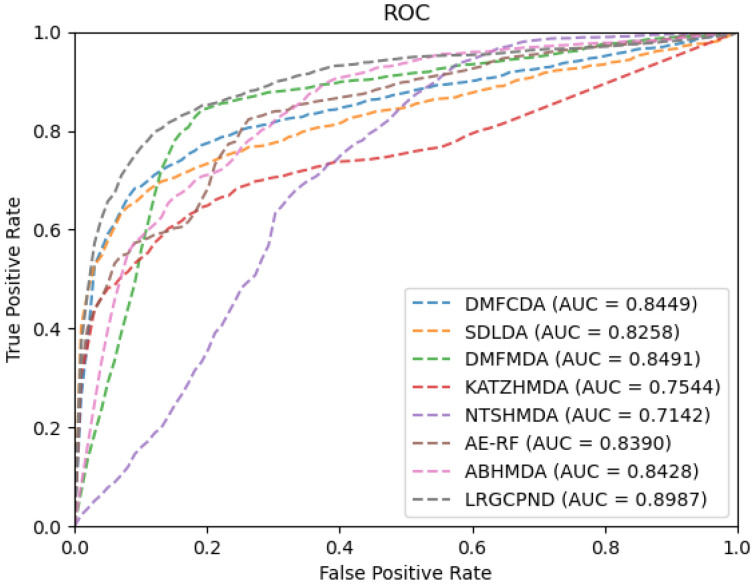
ROC curves of different methods on our dataset.

For the sake of rigor, we need to point out that since AE-RF [29] and ABHMDA [33] employ other similarity-based features besides the Gaussian interaction profile (GIP) kernel similarity. Considering the scarcity of relevant biological resources and convenience, we only calculated the GIP similarity for them in the experiments. Furthermore, the adjacency matrix allocated at the beginning of training is different, so the topology information of the interaction network needs to be re-extracted. We re-calculated the GIP similarity matrices during each cross-validation process for similarity-based methods, AE-RF, KATZHMDA [32], NTSHMDA [35], and ABHMDA. As plotted in Figure 5, it is evident that LRGCPND leads others with the average AUC value of 0.8987, which is 5.84% higher than the second-best method DMFMDA [34].

**Figure 6:**
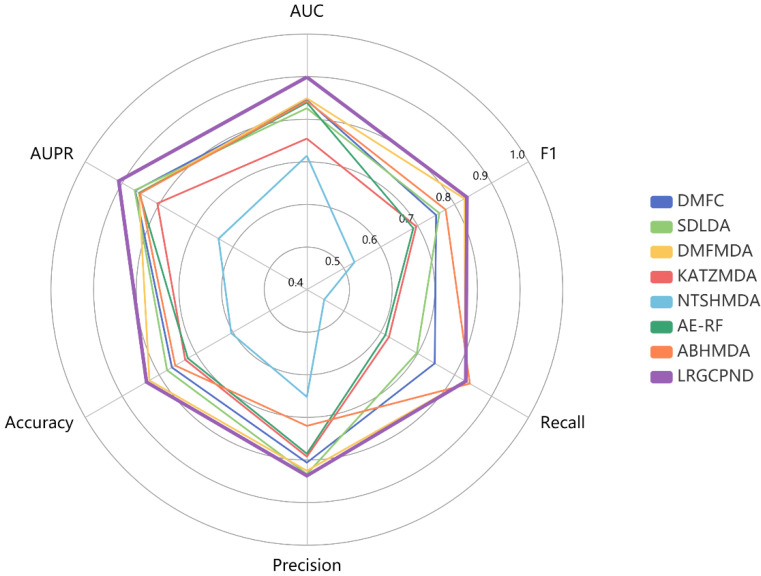
Performance comparison using multiple metrics in five-fold CV.

From statistics of various metrics listed in Table 4, except that the Recall value is slightly lower than ABHMDA, our model yields the optimal identification ability. Its AUPR, Accuracy, and F1 values achieve 0.9094, 0.8342, 0.8335, respectively. We also drew a radar chart to intuitively and comprehensively measure the capabilities of diverse models through various metrics, as shown in Figure 6. All six evaluation metrics range from 0.4 to 1.0. The farther the point from the center of the circle, the higher the value. It is also apparent to conclude that LRGCPND advantages over other methods.

These experimental results sufficiently demonstrate that our model is reliable and promising in inferring candidate ncRNA-drug resistance pairs.

### 2.6. Case Studies

The discovery of unknown associations between ncRNA and drug resistance matters tremendously for practical application. Thus, we selected two drugs, Cisplatin and Paclitaxel, and conducted case studies. Precisely, for a particular drug, to start with, we removed the known associated ncRNAs. Then, the remaining ncRNAs were sorted in descending order following the values predicted by LRGCPND. Lastly, we screened the top 15 ncRNAs and searched for supporting evidence in published literature.

Cisplatin is a common chemotherapeutic drug used to treat numerous cancers, including lung cancer, head and neck cancer, and ovarian cancer. Resistance frequently causes reduced efficacy of Cisplatin in chemotherapy [36]. Paclitaxel is another widely applied taxane medication. Chemoresistance to Paclitaxel makes its clinical application problematic [37]. Table 5 and Table 6 summarize the top 15 candidate ncRNAs of Cisplatin and Paclitaxel, respectively. We can see that 10 and 7 of the former and the latter are confirmed by existing evidence, indicating that our method has an excellent capability for predicting novel associated ncRNAs for drugs in terms of resistance. It is noteworthy that other unproven associations are likely to exist and deserve further relevant experiments.

## 3. Materials and Methods

### 3.1. Datasets

NoncoRNA: NoncoRNA [23] contains 5568 ncRNAs and 154 drugs in 134 cancers. This is the first database that provides diverse ncRNAs and associations between ncRNAs and drug resistance in cancers. We use the Feb 2020 version of the NoncoRNA database, which is publicly released at http://www.ncdtcdb.cn:8080/NoncoRNA (accessed on 10 March 2021).

ncDR: Hitherto, one of the most frequently used databases is ncDR [22] in the field of drug resistance-related non-coding RNA. Here, we adopt the data downloaded from the June 2016 version of the ncDR database. The dataset contains 5864 associations between ncRNAs and drug resistance, including 877 miRNAs and 162 lncRNAs from nearly 900 pieces of published literature. It now can be available on the website http://www.jianglab.cn/ncDR (accessed on 10 March 2021).

We manually integrated a set of 2693 associations between ncRNAs and drug resistance from NoncoRNA and ncDR datasets, including 625 ncRNAs and 121 drugs. Here we choose the experimental data. Besides, we clean the dataset by removing the redundant ones and associations in which a ncRNA only contains one drug resistance binding. The dataset can be expressed as:

(5) $$\mathbb{R}={\mathbb{R}}^{+}\cup {\mathbb{R}}^{-}$$

where ${\mathbb{R}}^{+}$ represents the positive dataset, which contains 2693 ncRNA-drug resistance associations verified with wet experiment. ${\mathbb{R}}^{-}$ represents the negative dataset, which contains a total of 72,932 ncRNA-drug resistance associations without verified experimentally. Earlier in Section 2.1, we have introduced the detail of sampling. Our dataset can be downloaded on the website https://github.com/TroyePlus/LRGCPND (accessed on 30 July 2021).

### 3.2. Problem Description

In order to predict the relationship between ncRNA and drug resistance, for a given set of m ncRNAs and n drugs, we use $U=\left\{u_{1},u_{2},\ldots ,u_{m}\right\}$ and $V=\left\{v_{1},v_{2},\ldots ,v_{n}\right\}$ respectively represent the collection of ncRNAs and drugs, and $R\in {\mathbb{R}}^{m\times n}$ is the correlation matrix. If ncRNA $u_{i}$ is related to drug resistance $v_{j}$, then the entry $R_{ij}=1$, otherwise $R_{ij}=0$. However, $R_{ij}=0$ does not mean that ncRNA $u_{i}$ has no association with the drug $v_{j}$. It may be that the relationship has not been found yet. In addition, we use $V_{i}^{+}=\left\{v_{j}|v_{j}\in V\right.\mathrm{and}\;\left.R_{ij}=1\right\}$ to represent the linked set of ncRNA $u_{i}$ found, and $V_{i}^{-}=V/V_{i}^{+}$ to represent the non-linked set. $D=\left\{\left(u_{i},v_{j}\right)|R_{ij}=1\right\}$ is defined as the set of all linked ncRNA and drug resistance pairs.

### 3.3. Graph Construction

We use a bipartite graph $G\left(U,V,E\right)$ to express the associations between different ncRNAs and drug resistance, where U, V are the previously defined ncRNA set and drug set. Every edge e belonging to E represents a verified association between ncRNA u and drug resistance v.

### 3.4. Graph Embedding

Matrix factorization is a common method of graph embedding. Matrix factorization only uses the linear relationship between entities and can be applied to data that only contains associations. However, the matrix factorization method cannot make full use of data information, and its ability to extract high-order features is weak. In recent years, graph-based models have become popular in the field of semi-supervised classification. The network built by graphs combined with deep learning methods can be applied to graph embedding to obtain vector representations of graphs or graph nodes [38]. Graph convolutional neural network is often used in the field of association prediction in biological information. The design of graph convolutional neural network is inspired by convolutional neural network, which is widely used in the field of computer vision. Its advantage is that it can extract the structural features of node neighborhoods and then learn higher-order relationships. But obvious disadvantages are the over-smoothing problem and time-consuming calculation. In this work, the task of ncRNA-drug resistance association is similar to the recommendation problem, where ncRNA corresponds to the user, and the drug resistance is equivalent to the project. The verified association is equal to the user’s viewing/shopping history. Therefore, the graph convolutional neural network method, which is very popular in the recommendation task, can be applied to our problem. Here, we solve the above problems with linear propagation and residual block based on GCN. We first construct the adjacency matrix A of the bipartite graph G as follows:

(6) $$A=\left[\begin{array}{cc} 0 & R\\ R^{T} & 0 \end{array}\right]$$

Then use E to represent the embedding matrix of ncRNA and drug resistance. We generate initial values from the normal distribution given standard deviation = 0.1 to fill the initial embedding matrix with nn.initial.normal. Every epoch in training, LRGCPND treats the embedding matrix as input:

(7) $$E^{0}=E$$

where E is calculated in each iteration and will be updated.

### 3.5. Feature Aggregation

There is no intra-domain edge in the bipartite graph, so the message passing and node feature aggregation are only performed through the inter-domain edge for the convolution of the bipartite graph. We use the spectral rule to aggregate feature of graph:

(8) $$\begin{array}{ll} f_{agg}{\left(\;\tilde{A},E\right)}_{i} & ={\;\tilde{D}}^{-0.5}{\;\tilde{A}}_{i}{\;\tilde{D}}^{-0.5}E\\ & =\sum_{k=1}^{N}{\;\tilde{D}}_{i,k}^{-0.5}\sum_{j=1}^{N}{\;\tilde{A}}_{i,j}\sum_{l=1}^{N}{\;\tilde{D}}_{j,l}^{-0.5}E_{j}\\ & =\sum_{j=1}^{N}{\;\tilde{D}}_{i,i}^{-0.5}{\;\tilde{A}}_{i,j}{\;\tilde{D}}_{j,j}^{-0.5}E_{j} \end{array}$$

where $\;\tilde{A}=A+I$, I is the identity matrix. $\;\tilde{D}$ is the degree matrix of $\;\tilde{A}$. As is adopted widely in GCN, spectral rule considers not only the degree of ith node, but also the degree of the jth node when calculating the aggregation of the ith node.

### 3.6. Linear Transition

We remove the nonlinear transformation functions at the end of each layer. Despite the linear propagation of LRGCPND, the “receptive field” of our model is the same as a K-layer GCN. The k+1 step embedding could be calculated as:

(9) $$E^{k+1}=f_{agg}\left(\;\tilde{A},E^{k}\right)W^{k}$$

where $W^{k}$ represents the linear transformation, $E^{k}$ is the k step embedding.

Due to the linear transformation, we can get the matrix form to model each ncRNA n’s and drug-resistance r’s embedding:

(10) $${\left[E^{k+1}\right]}_{n}=e_{n}^{k+1}=\left[\frac{1}{d_{n}}e_{n}^{k}+\sum_{i\in R_{n}}\frac{1}{d_{i}\times d_{n}}e_{i}^{k}\right]W^{k}$$

(11) $${\left[E^{k+1}\right]}_{r}=e_{r}^{k+1}=\left[\frac{1}{d_{r}}e_{r}^{k}+\sum_{j\in R_{r}}\frac{1}{d_{r}\times d_{j}}e_{n}^{k}\right]W^{k}$$

where d is the diagonal degree of ncRNA n(drug-resistance r) in G. $R_{n}$($R_{r}$) represents the neighbors of node n (r) in G.

### 3.7. Residual Block in LRGCPND

In a graph convolution network, there is an over-smoothing problem caused by network stacking. The role of GCN is equivalent to low-pass filtering, making the input signal smoother, which is an inherent advantage of the GCN model. However, after multiple executions of GCN operations, the signals will tend to be the same, so the diversity of node characteristics is lost, which is a fatal disadvantage for tasks related to node classification. From the perspective of the spectral domain, analyzing the frequency response function of GCN points out that if the smoothing operation is continuously performed on a graph signal, the graph signal will eventually become equal everywhere, ultimately losing the discrimination information between nodes. Here we adopt the residual block [39] proposed by Kaiming He to establish identity mapping. The output of our model can be described as:


(12) $${\hat{o}}_{nr}^{k+1}={\hat{o}}_{nr}^{k}+e_{n}^{k+1}\cdot e_{r}^{k+1}$$

### 3.8. Model Optimization

BPR [40] is a sorting algorithm based on matrix decomposition. It is not a global scoring optimization but a sorting optimization for each ncRNA’s related drug-resistance preferences. It is a pairwise sorting algorithm. For each triple <n,i,j>, the model hopes to make the ncRNA n’s difference between drug-resistance i and j more obvious.

(13) $$\underset{\Theta }{\mathrm{minL}}=\sum_{a=1}^{M}\sum_{(i,j)\in D_{a}}\left(-\ln\left(s\left({\hat{o}}_{ai}-{\hat{o}}_{aj}\right)\right)\right)+\lambda \|\Theta_{1}\|{}^{2}+\lambda \|\Theta_{2}\|{}^{2}$$

where $\Theta_{1}=\left[E^{0}\right]$, $\Theta_{2}=\left[W\right]$, E is updated after the model backward propagation. λ controls the strength of $L_{2}$ regularization. $R_{a}$ denotes the positive subset for a of drug set V. $D_{a}=\left\{\left(i,j\right)|i\in R_{a}\wedge j\in V-R_{a}\right\}$ represents the pairs containing positive sample i and negative sample j.

## 4. Conclusions

Drug resistance response has caused vital challenges to clinical treatment. Numerous studies have indicated that ncRNA plays a pivotal role in the mechanisms of drug resistance. Accurately identifying the ncRNA-drug resistance association pairs is conducive to drug development and promotes clinical treatment. In this work, we propose LRGCPND, a graph convolutional network computational framework for mining the latent associations between ncRNA and drug resistance through linear transition and residual prediction. To our best knowledge, this is the first computational prediction approach in this field. We represent the relationship between ncRNA and drug resistance in a bipartite graph and exploit limited information to learn complex latent factors for edge prediction. LRGCPND first captures the neighborhood representations by aggregation. Then, it performs feature transformation through linear operations. Finally, the embedding vectors of convolutional layers are concatenated through residual blocks to achieve prediction.

Experimental results and case studies corroborate the effectiveness of our model, to which several aspects may contribute. We utilize graph convolution to perform relatively more adequate representation learning on the original association data with inadequate information. Residual blocks enable the model to attain higher-layer potential characteristics, and linear feature propagation keeps the model lightweight and flexible to extend to datasets on a large scale. In conclusion, our model is promising and facilitates further research in predicting novel associated ncRNAs for drug resistance. Our study helps build a systematic map of ncRNA and drug resistance, provides more insights into drug resistance, and aids in identifying effective therapeutic combinations.

As with many computational prediction methods, LRGCPND also has its limitations. First, LRGCNPND only utilizes ncRNA-drug resistance association data. The quality and coverage of the association data would affect the performance. Second, LRGCPND makes predictions with ncRNAs containing subtypes. Despite this provides insights from a broader perspective, differences between subtypes would cause bias. In the future, we will combine the attention mechanism and integrate multiple heterogeneous data to improve the performance further.

## Figures and Tables

**Table 1:** Prediction results of LRGCPND in five-fold CV.

No.	AUC	AUPR	Acc.	P.	R.	F1
1	0.9052	0.9149	0.8467	0.8410	0.8550	0.8479
2	0.8961	0.8949	0.8383	0.8473	0.8253	0.8362
3	0.8946	0.9068	0.8262	0.8395	0.8067	0.8227
4	0.8986	0.9116	0.8290	0.8254	0.8346	0.8299
5	0.8989	0.9186	0.8309	0.8315	0.8299	0.8307
Avg.	0.8987	0.9094	0.8342	0.8369	0.8303	0.8335

**Table 2:** Prediction results of LRGCPND with different depth *K*.

*K*	AUC	AUPR	Acc.	P.	R.	F1
1	0.8961	0.9059	0.8268	0.8290	0.8236	0.8262
2	0.8946	0.9038	0.8273	0.8353	0.8158	0.8252
3	0.8942	0.9054	0.8303	0.8337	0.8255	0.8294
4	0.8987	0.9094	0.8342	0.8369	0.8303	0.8335
5	0.8855	0.8947	0.8203	0.8210	0.8195	0.8201

**Table 3:** Prediction results of LRGCPND with different embedding size *S*.

*S*	AUC	AUPR	Acc.	P.	R.	F1
8	0.8906	0.8930	0.8167	0.7962	0.8515	0.8228
16	0.8915	0.8937	0.8234	0.8135	0.8396	0.8262
32	0.8987	0.9094	0.8342	0.8369	0.8303	0.8335
64	0.8874	0.8974	0.8275	0.8502	0.7950	0.8216
128	0.8801	0.9006	0.8184	0.8597	0.7612	0.8073

**Table 4:** Prediction results of different methods in five-fold CV.

Methods	AUC	AUPR	Acc.	P.	R.	F1
DMFCDA	0.8449	0.8649	0.7654	0.8062	0.7463	0.7499
SDLDA	0.8258	0.8663	0.7785	0.8330	0.6978	0.7588
DMFMDA	0.8491	0.8546	0.8264	0.8253	0.8288	0.8269
KATZMDA	0.7544	0.8048	0.7295	0.7921	0.6223	0.6964
NTSHMDA	0.7142	0.6391	0.6047	0.6518	0.4470	0.5289
AE-RF	0.8390	0.8535	0.7223	0.7853	0.6127	0.6881
ABHMDA	0.8428	0.8516	0.7565	0.7199	0.8413	0.7756
LRGCPND	0.8987	0.9094	0.8342	0.8369	0.8303	0.8335

**Table 5:** The top 15 miRNAs related to Cisplatin resistance predicted by LRGCPND.

Rank	ncRNA	Evidence (PMID)
1	miR-30a	28222434
2	miR-140	31288529
3	miR-361	33531993
4	miR-660	Unconfirmed
5	miR-151	Unconfirmed
6	miR-103	31372241
7	miR-212	Unconfirmed
8	miR-30c	29440633
9	miR-324	31778188
10	miR-1183	Unconfirmed
11	miR-497	26238185
12	miR-122	31152437
13	miR-625	Unconfirmed
14	miR-425	31632022
15	miR-342	32397872

**Table 6:** The top 15 miRNAs related to Paclitaxel resistance predicted by LRGCPND.

Rank	ncRNA	Evidence (PMID)
1	HOTAIR	32743678
2	miR-19b	Unconfirmed
3	miR-26b	30899303
4	miR-10b	Unconfirmed
5	miR-152	32913475
6	miR-20a	Unconfirmed
7	miR-212	32774496
8	miR-339	28940895
9	miR-424	Unconfirmed
10	miR-103	Unconfirmed
11	miR-21-5p	31169019
12	miR-628	Unconfirmed
13	CRNDE	32581554
14	miR-151	Unconfirmed
15	miR-543	Unconfirmed

## Data Availability

The code and data of this study are available at https://github/TroyePlus/LRGCPND (accessed on 30 July 2021).
